# MIGGRI: A multi-instance graph neural network model for inferring gene regulatory networks for Drosophila from spatial expression images

**DOI:** 10.1371/journal.pcbi.1011623

**Published:** 2023-11-08

**Authors:** Yuyang Huang, Gufeng Yu, Yang Yang

**Affiliations:** Department of Computer Science and Engineering, Shanghai Jiao Tong University, and Key Laboratory of Shanghai Education Commission for Intelligent Interaction and Cognitive Engineering, Shanghai, China; University of Pittsburgh, UNITED STATES

## Abstract

Recent breakthrough in spatial transcriptomics has brought great opportunities for exploring gene regulatory networks (GRNs) from a brand-new perspective. Especially, the local expression patterns and spatio-temporal regulation mechanisms captured by spatial expression images allow more delicate delineation of the interplay between transcript factors and their target genes. However, the complexity and size of spatial image collections pose significant challenges to GRN inference using image-based methods. Extracting regulatory information from expression images is difficult due to the lack of supervision and the multi-instance nature of the problem, where a gene often corresponds to multiple images captured from different views. While graph models, particularly graph neural networks, have emerged as a promising method for leveraging underlying structure information from known GRNs, incorporating expression images into graphs is not straightforward. To address these challenges, we propose a two-stage approach, MIGGRI, for capturing comprehensive regulatory patterns from image collections for each gene and known interactions. Our approach involves a multi-instance graph neural network (GNN) model for GRN inference, which first extracts gene regulatory features from spatial expression images via contrastive learning, and then feeds them to a multi-instance GNN for semi-supervised learning. We apply our approach to a large set of *Drosophila* embryonic spatial gene expression images. MIGGRI achieves outstanding performance in the inference of GRNs for early eye development and mesoderm development of *Drosophila*, and shows robustness in the scenarios of missing image information. Additionally, we perform interpretable analysis on image reconstruction and functional subgraphs that may reveal potential pathways or coordinate regulations. By leveraging the power of graph neural networks and the information contained in spatial expression images, our approach has the potential to advance our understanding of gene regulation in complex biological systems.

## Author summary

Spatial expression images are valuable resources for reconstructing gene regulatory networks (GRNs) due to their rich spatio-temporal patterns. However, computational methods for image-based GRN inference are still in the early stages of development. In this study, we aim to extract comprehensive information from multiple images for each gene and utilize known GRN to infer unknown interactions.

To achieve this, we implement contrastive learning to extract gene regulatory features from spatial expression images and also develop a multi-instance graph neural network model, MIGGRI, for GRN inference. Our approach demonstrates good performance in GRN inference for *Drosophila* eye and mesoderm development based on spatial expression patterns.

Compared to previous methods, MIGGRI exhibits high accuracy, robustness, and interpretability. Especially, MIGGRI can handle missing data and identify crucial regulators and pathways involved in the regulation process of *Drosophila* development. Our findings provide a promising approach for image-based GRN inference and shed light on the complex regulatory mechanisms underlying development processes.

## Introduction

Reverse engineering gene regulatory networks (GRNs) has been a central task in systems biology, as it provides a comprehensive understanding of the interplay between transcription factors (TFs) and their target genes. Traditionally, the task of GRN inference was based on gene expression data obtained from microarray technology [1]. However, with the rapid development of high-throughput technologies, such as RNA-sequencing, these have become the major data source for GRN inference [2].

A lot of computational methods have been proposed based on various statistical methods and machine learning models, including linear regression [3], mutual information [4], Pearson’s and Spearman’s correlation [5], Bayesian networks [6], and Gaussian graphical models [7]. While methods based on gene co-expression often infer influential GRNs, where the networks consist of indirect interactions, transcription factor (TF) binding data has been leveraged to uncover physical/direct interactions, including ChIP-Chip or ChIP-Seq data. In addition, the advances in multi-omics experiments have enabled the integration of various types of data, such as transcriptomics, proteomics, interactomics, and epigenomics, to enhance the robustness and scalability of GRN inference [8]. By leveraging these various sources of data, researchers can gain a more comprehensive understanding of gene regulation and the underlying mechanisms in complex biological systems.

Despite current progress, the inference of GRNs has remained an extremely challenging task in bioinformatics. For one thing, most of the existing methods are incompetent to handle large-scale networks; for another thing, the inference performance is largely limited by the input data quality, due to the missing values and high noise in gene expression. More importantly, since the distribution of gene expression in space is crucial for identifying co-expression patterns, the average expression levels obtained by microarray or RNA-seq often fail to reflect true interactions between genes.

In recent years, with the rapid development of spatial transcriptomics, especially various *in situ* hybridization (ISH) technologies for visualizing gene expression distribution, like RNA ISH [9], SeqFISH [10], and MERFISH [11], methods based on spatial expression data have emerged [12]. As spatial data delineates complex spatio-temporal regulation patterns, the network reconstruction can benefit more from the spatial expression data [13]. Early works utilizing spatial expression are mainly unsupervised, which predict gene interactions based on certain similarities or correlations. For instance, the GINI method flattened the intensity of each pixel of gene expression image in *Drosophila* embryo as a feature vector and adopted a multi-variate Gaussian model to build the gene interaction network [14]; staNMF implemented a non-negative matrix factorization method to identify the principal patterns of expression images and inferred gene-gene interaction based on their similarity computed by the principal patterns [15]. Due to the lack of supervision, the similarity or correlation-based methods may identify indirect interactions rather than direct regulatory relations. In addition, acquiring spatial expression images of a gene involves gathering data from multiple individuals, each with varying views. As a result, the images exhibit significant variations across different samples, and the number of images per gene may differ, making it a challenging multi-instance learning problem. Extracting effective tissue expression information features from these multi-instance images remains a significant challenge in the field.

Benefiting from the increasingly revealed GRNs and deep learning techniques, supervised deep neural networks have been employed in GRN reconstruction [16–18]. GripDL [17] concatenates two images (from a TF and a target gene respectively) and employs a single ResNet [19] to predict the existence of regulatory interaction; while ConGRI [18] adopts a siamese network [20] to learn the image-pair input. Li et al. proposed SDINet to fuse RNA-seq data and gene expression image data [16]. However, these methods have some limitations. First, they perform pairwise prediction without considering the GRN topology, i.e., the model input consists of only the expression data of a gene pair. Second, they are unable to predict gene pairs for which expression data is missing. Furthermore, the multi-instance problem has not been effectively resolved yet.

Recently, the development of graph neural networks [12, 16] has greatly enhanced the performance of the prediction tasks with graph-structured data. Instead of taking a pair of gene features as input, GNNs learn from graphs, including both node features and graph topological information. Thus, GNNs have become a good choice for modeling gene interaction networks, and a few GNN-based GRN inference methods have been proposed [12, 16, 21]. Especially, with the rapid accumulation of single-cell data, gene expression in single cells has been used to improve the node features in graphs [12, 16]. However, GNN-based methods for spatial expression data are relatively few. The major reason is that RNA-seq and scRNA-seq yield quantitative data and allow high-throughput assaying for whole genomes, which are more suitable for correlation-based gene interaction identification; while spatial expression data is often in a multi-instance format involving high-resolution microscopic images with high complexity, large sizes, low quantitative power, and low throughput. Extracting effective image features that preserve spatial expression patterns and integrating them into the prediction model is a challenging task.

In this study, we predict gene regulatory interactions directly based on spatial expression images, considering that the spatio-temporal gene expression patterns involved in these images are indispensable to accurately infer gene regulatory networks. Although without resolved quantitative values, the expression levels and spatial patterns represented in the form of pixel intensities and their distribution in images can be abundant information resources. Here we propose a method called MIGGRI, a **M**ulti-**I**nstance **G**raph neural network model for **G**ene **R**egulatory network **I**nference, which consists of two major components: 1) a contrastive learning-based feature extractor for expression images, and 2) a multi-instance GNN model to perform the network inference and address the multi-instance issue (each gene corresponds to multiple expression images). We evaluate MIGGRI on two GRNs of *Drosophila*, specifically related to eye development and mesoderm development. Our assessment is based on a large collection of *Drosophila* embryonic spatial gene expression images, which constitute one of the largest datasets available from the genome projects of model organisms. The contributions of this work can be summarized as follows.

- We propose a GNN model, MIGGRI, for GRN inference using spatial expression images. Equipped with a contrastive learning-based feature extractor and a multi-instance aggregator, MIGGRI effectively captures gene regulation information from images and considers expression patterns from multiple images comprehensively.
- MIGGRI achieves outstanding performance on large-scale GRN inference, and outperforms the deep learning models designed for regulatory interaction prediction by a large margin. Besides, it can identify important links in the networks and the subgraphs composed of these links reveal pathways involved in the regulation process.
- We evaluate the model’s ability to handle missing data and find that it exhibits a high degree of tolerance towards genes without spatial expression information. Moreover, our analysis of the model’s ability to reconstruct missing images indicates that the gene representations learned by the model capture valuable spatial expression patterns present in expression images.

## Materials and methods

### Gene regulatory interaction data

This research is conducted on two GRNs of *Drosophila* that are involved in the development of the eye and mesoderm, respectively. For the *Drosophila* eye development dataset, we construct a partial GRN according to the study of [22] as the training data. The known regulatory interactions were validated by both co-expression relationships (by RNA-Seq) and physical interactions (using computational motif inference), and we only use the high-confident interactions (drawn from direct evidence) in the training to ensure the reliability of the labels. This partial network is very sparse, whose density is 0.55%, suggesting that a lot of regulatory links may remain unknown. (The density is computed by $\frac{2|E|}{\left|V|(|V|-1\right)}$, where |E| is the number of edges, and |V| is the number of nodes in the graph.) Compared to the eye development GRN, the size of the mesoderm development network is much smaller, where the known regulatory interactions are verified by ChIP-chip experiments [23].

### Spatial expression data

**Fig 1:**
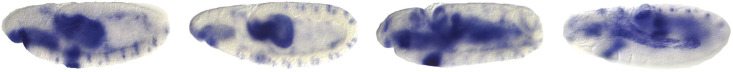
The expression images of gene *CG10002* from developmental stage 11–12.

The spatial expression data are from the Berkeley *Drosophila* Genome Project (BDGP) [24, 25], which houses the largest number of digital images of gene expression patterns in *Drosophila* embryogenesis by RNA *in situ*, including 8591 genes and over 14000 ISH images at different developmental stages of *Drosophila* embryos. When performing hybridization operations to obtain gene expression patterns, probes are designed, added, and hybridized to only one target gene at a time, followed by staining or other operations. Thus, each image records the expression distribution for a single gene. Moreover, each gene may correspond to a set of images captured from different views, i.e., lateral, ventral, and dorsal. To ensure image quality, we extract standardized images from the FlyExpress database [26–28], where the images are further processed (cropping, aligning, and scaling to the size of 320×128). Fig 1 displays some examples of expression images for gene CG10002. In these images, pixel intensity corresponds to the expression level of the gene, with darker areas indicating higher expression levels.

In the BDGP dataset, the development process of *Drosophila* is divided into 16 specific stages, which are further classified into 6 phases. Note that the study of [22] identified the eye development GRN using samples from fruit fly larvae, while the eye development begins early in the embryo (a lot of genes are annotated by eye-related terms in BDGP). Thus we use the images from the last phase range of the embryonic period, which corresponds to stage 13–16 in BDGP’s development stage division. Our experiments demonstrate that the prediction results based on the spatial expression from the last developmental stage of the embryo are highly consistent with the labeled data, achieving over 80% accuracy for the eye development dataset and over 70% accuracy for the mesoderm development dataset.

### Construction of training and test sets

To assess the performance of MIGGRI, for both GRNs, we randomly select 20% of the known interactions as the positive interactions in the test set, and the remaining 80% of the interactions are divided into the training and validation set with a ratio of 9:1. The negative data is randomly sampled from gene pairs that are not connected in the known network, meaning that it has no overlap with the high- or medium-confidence links (These randomly sampled pairs may still have unknown interactions and may not necessarily represent true negatives). The positive-to-negative ratio is set to 1:1 in both the training set and test set. Statistics of the datasets are shown in S1 and S2 Tables.

### Problem formulation

This study aims to reconstruct large-scale gene regulatory networks (GRNs) based on gene spatial expression data and partial network topology. A GRN can be denoted by a graph G=(V,E), where V is the set of nodes, i.e. genes, and E is the set of edges, i.e. gene-gene interactions.

Due to the multi-instance nature, a gene corresponds to a set of expression images $I_{i}=\left\{I_{i}^{\text{V}},I_{i}^{\text{D}},I_{i}^{\text{L}}\right\}$, where $I_{i}^{\text{V}}$, $I_{i}^{\text{D}}$, and $I_{i}^{\text{L}}$ denote three subsets of images captured at the orientation of ventral, dorsal, and lateral, respectively. In our model, the node representation **x**_*i*_ of the *i*th node is generated by first a CNN-based feature extractor and then an aggregator (Eq (1)),

$$\begin{array}{c} x_{i}=\text{Aggregate}\left(\text{CNN}\left(I_{i}\right)\right) \end{array}$$ (1)

The inference of GRNs is essentially a link prediction task in graphs. In this study, we perform supervised learning to identify unknown interactions, i.e. to predict whether there is an edge existing between two given nodes based on the existing edges in the graph. Let **X** and **A** be the node feature matrix and adjacency matrix, respectively. Through the GNN training, we can get the updated node feature matrix **X′**, i.e.,

$$\begin{array}{c} X^{\prime }=\text{GNN}\left(X,A\right). \end{array}$$ (2)

Thus, an updated adjacency matrix **A′** can be inferred according to **X′**. In this way, all potential interactions could be revealed.

### Model overview

The proposed MIGGRI exploits both gene spatial expression information and known regulatory interactions to predict unknown interactions. Gene regulatory networks can be naturally modeled as graphs where nodes are genes and links between genes denote their interactions. Thus the prediction module adopts a GNN as the backbone network. Since incorporating image information into GNN is not straightforward and to ensure high-quality initial node embeddings for the graph, we propose a two-stage learning scheme. The first stage aims to extract features from gene expression images. One challenge of this stage is to preserve regulatory interaction-related features from high-dimensional image data in a limited supervision scenario (only a portion of regulatory relations is known). To address this challenge, we adopt a siamese convolutional network to learn the pairwise interaction information of the expression images, whose input is a pair of images (from a TF and a target gene, respectively) and the target output is a binary label (1 for interaction and 0 for no interaction). Importantly, as genes have multiple instances of spatial expression images, the pairwise setting allows us to construct a large number of input pairs that exceed the number of known regulatory relations. This provides more variants for MIGGRI to capture the regulatory-related features. By optimizing the contrastive loss, the convolutional layers can be trained to extract image features that are correlated to regulatory interactions. Then, in the second stage, a multi-instance GNN is designed to aggregate the multi-instance features with graph topology information. MIGGRI takes both the node features and the adjacent matrix denoting links in the graph as input. The adjacent matrix can be directly generated by known regulatory interactions, while the gene representation is learned from gene spatial expression data before training the GNN.

**Fig 2:**
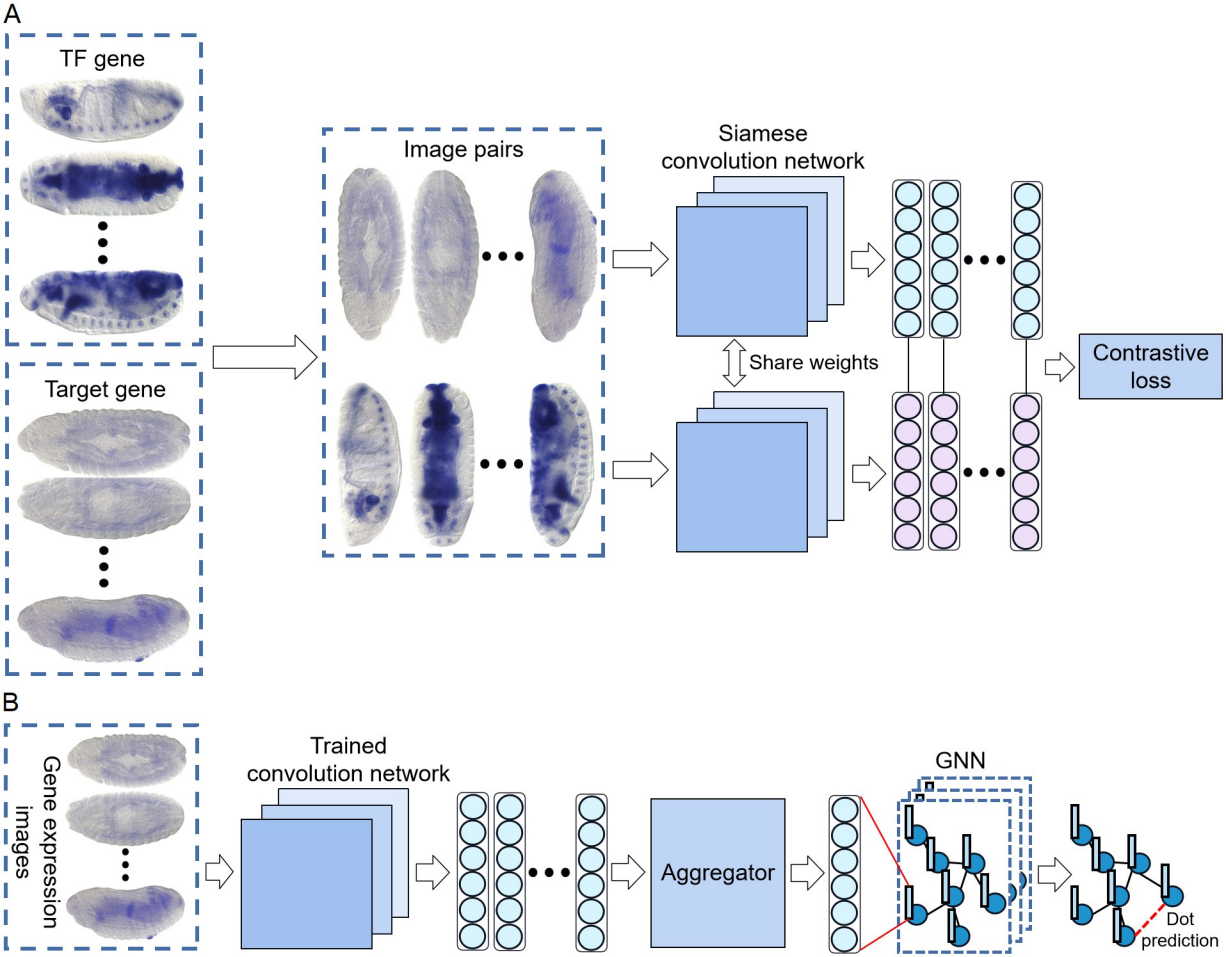
Overview of MIGGRI. (A) Stage I: Image embedding extraction via a siamese convolution network learned by contrastive loss. The gene expression images of *Drosophila* embryos were captured at different orientations (ventral, dorsal, and lateral), thus the input two images should be in the same orientation to avoid differences caused by views. (B) Stage II: GRN inference via the multi-instance graph neural network. For each gene, its multiple image embeddings are first aggregated into a single feature vector that serves as the node embedding in the graph. During the training procedure, the node embeddings are updated. Finally, unknown links between pairs of genes are predicted by the dot product operation on their node embeddings.

Fig 2A shows the network architecture for Stage I. Note that each gene may contain multiple expression images captured at different orientations (ventral, dorsal, and lateral), the input two images should be in the same orientation to avoid differences caused by views. After training, each image is passed through the CNN to yield an embedding vector. Then, each gene may correspond to multiple embedding vectors, which are aggregated into a single representation in Stage II. As shown in Fig 2B, there is an aggregator that yields node embeddings for the downstream GNN. Through training the GNN, node embeddings get updated, and finally we use a decision module to infer gene interactions according to the dot product of the final embeddings of two genes.

We describe the two stages in detail in the following sections.

### Stage I: Extracting ISH image features with a siamese convolution network

CNNs have been widely adopted to learn features from image data, by fitting functions from input images to output labels. In this study, the target output is the existence of regulatory interaction between two genes. Thus, we adopt a siamese network structure to allow pairwise input.

Specifically, an input pair is denoted by $P=\langle I_{i}^{p,o},I_{j}^{q,o}\rangle$, where $I_{i}^{p,o}$ and $I_{j}^{q,o}$ denote the *p*th image from the *i*th gene and the *q*th image from the *j*th gene, respectively, which have the same orientation *o* (*o* ∈ {V, D, L}). Let the label of *P* be *y*_*i*,*j*_ (1 for regulatory relation and 0 for no regulatory relation). Apparently, for a single pair of genes, there may be multiple input pairs of images with the same label. Following the previous works [17, 18], negative samples are randomly selected from the gene pairs which are not connected in the known GRN.

Benefiting from the pairwise input setting, the training examples are greatly increased compared to single-image input, which enables sufficient training for the CNN layers.

Here we use a modified VGG-16 as the backbone CNN model, and the siamese network is trained with the contrastive loss *L*_*c*_ as shown in Eq (3) [29],

$$\begin{array}{c} \begin{array}{c} L_{c}=\{\begin{array}{c} \frac{1}{2}\left|\right|x_{i}^{p,o}-x_{j}^{q,o}{\left|\right|}_{2}^{2},\;\text{if}\;y_{i,j}=1\\ \frac{1}{2}\text{max}(0,m-||x_{i}^{p,o}-x_{j}^{q,o}{\left|\right|}_{2}{)}^{2},\;\text{if}\;y_{i,j}=0 \end{array}, \end{array} \end{array}$$ (3)

where *m* denotes the distance margin, a predefined hyperparameter. Through contrastive learning, the model will pull the image feature embeddings close for the interacting pairs of genes while push away those non-interacting pairs.

### Stage II: Multi-instance graph neural network

Given the image embedding vectors learned in Stage I, we first design an aggregator to convert the set of image embeddings for a gene to a single gene representation (as formulated in Eq (1)). There are three alternative aggregators, based on mean pooling, max pooling, and a long short-term memory (LSTM) network with random shuffling. The first two aggregators have no parameters, which just take the mean or max value per dimension over the given set of image vectors; while the LSTM is a special type of recurrent neural network to learn representation for the input sequence. As our input is a set instead of a sequence, a random shuffling strategy is adopted to ensure order invariance, i.e. in each training epoch, the order of image embeddings in the set is randomly shuffled.

Then we use a two-layer GNN to learn from the aggregated feature for each gene node and the partially known network structure. We employ the GraphSAGE-mean [30] as the backbone network. Unlike the two other popular GNN models, GCN and GAT, GraphSAGE-mean involves a crucial step of taking the mean operation on the embeddings over the neighborhood for a given node. As a result, GraphSAGE is particularly well-suited for semi-supervised learning tasks, especially when some node features are missing. In our experiments, GraphSAGE-mean shows better performance than GCN and GAT. The comparison of different backbone networks is provided in S4 Table. The message passing and aggregating mechanism is formulated in Eq (4).

$$\begin{array}{c} \begin{array}{cc} & h_{N(i)}^{\left(l+1\right)}=\text{MEAN}\left(\left\{h_{j}^{\left(l\right)},\forall j\in N\left(i\right)\right\}\right)\\ & h_{i}^{\left(l+1\right)}=\sigma (W^{\left(l\right)}\cdot \text{CONCAT}(h_{i}^{\left(l\right)},h_{N(i)}^{\left(l+1\right)})), \end{array} \end{array}$$ (4)

where $h_{i}^{\left(l\right)}$ is the feature vector of the *i*th node in the *l*th layer. N(i) is the set of neighbors of gene *i* and ***W***^(*l*)^ is the weight matrix of the *l*th layer. MEAN(⋅) is an operator that computes the average vector for a set of vectors, and *σ* denotes the activation function.

After training the GNN, we adopt the dot product of two gene embeddings ($h_{i}^{out}$ and $h_{j}^{out}$) to predict whether the interaction exists, as described in Eq (5).

$$\begin{array}{c} \begin{array}{c} {\hat{y}}_{ij}=\sigma \left(h_{i}^{out}\cdot h_{j}^{out}\right). \end{array} \end{array}$$ (5)

## Results

### Experimental settings

In Stage I, we optimize the siamese convolution network by Adam optimizer [31] with a learning rate of 5e-6. The distance margin *m* in the contrastive loss is set to 1, which is a common choice in previous studies [18, 32]. For the eye development GRN, the model is trained for 50 epochs with batch size 16. For the mesoderm development GRN, the model is trained for 22 epochs with batch size 32.

In Stage II, we train the LSTM-based aggregator and GraphSAGE jointly. We use the Adam optimizer with a learning rate of 5e-2. The decision threshold for the existence of interaction is set to 0.5. Based on the best performance on the validation set, the model on eye development dataset is trained for 78 epochs, and the model on mesoderm development dataset is trained for 3 epochs.

### MIGGRI achieves promising performance for GRN inference

We compare our method with four baseline models, namely staNMF [15], SIFT_LoR [33–35], GripDL [17], and ConGRI [18]. Details are listed below.

- **StaNMF**: an unsupervised learning method that extracts principal patterns from gene expression images based on nonnegative matrix factorization and selects the set of principal patterns with a stability criterion;
- **SIFT_LoR**: a supervised learning method using SIFT descriptor [33] and logistic regression;
- **GripDL**: a deep CNN model that performs binary classification based on combined gene pair images;
- **ConGRI**: a deep learning model that employs a siamese network for feature extraction and multi-layer perceptron (MLP) for prediction.

The first baseline is an unsupervised model, the second one is a traditional machine learning method with classic feature extraction, and the remaining ones are deep learning methods. Especially, GripDL and ConGRI adopt CNN to extract image features but do not leverage network topology. The implementation of staNMF, GripDL, and ConGRI are publicly available. We follow the original implementations by directly using their source codes, and these methods were all primarily designed for the reconstruction of regulatory networks using spatial gene expression images. SIFT_LoR uses scale-invariant feature transform (SIFT) to extract image features, which is a classical algorithm in computer vision (CV) with a standard implementation. We adopt the hyperparameter settings provided in the original implementations of staNMF, GripDL, and ConGRI, and well-tune the hyperparameters of SIFT_LoR to ensure consistency and comparability.

We use two common metrics, accuracy and F_1_ score (formulated in Eq (6)), to assess the performance.

$$\begin{array}{c} \begin{array}{cc} & \text{Accuracy}=\frac{\text{TP}+\text{TN}}{\text{TP}+\text{FP}+\text{TN}+\text{FN}},\\ & {\text{F}}_{1}\;\text{score}=\frac{2\times \text{TP}}{2\times \text{TP}+\text{FP}+\text{FN}}, \end{array} \end{array}$$ (6)

where TP, FP, TN, and FN correspond to the number of true positives (correctly predicted interaction), false positives (incorrectly predicted interaction), true negatives (correctly predicted non-interaction), and false negatives (incorrectly predicted non-interaction), respectively.

**Fig 3:**
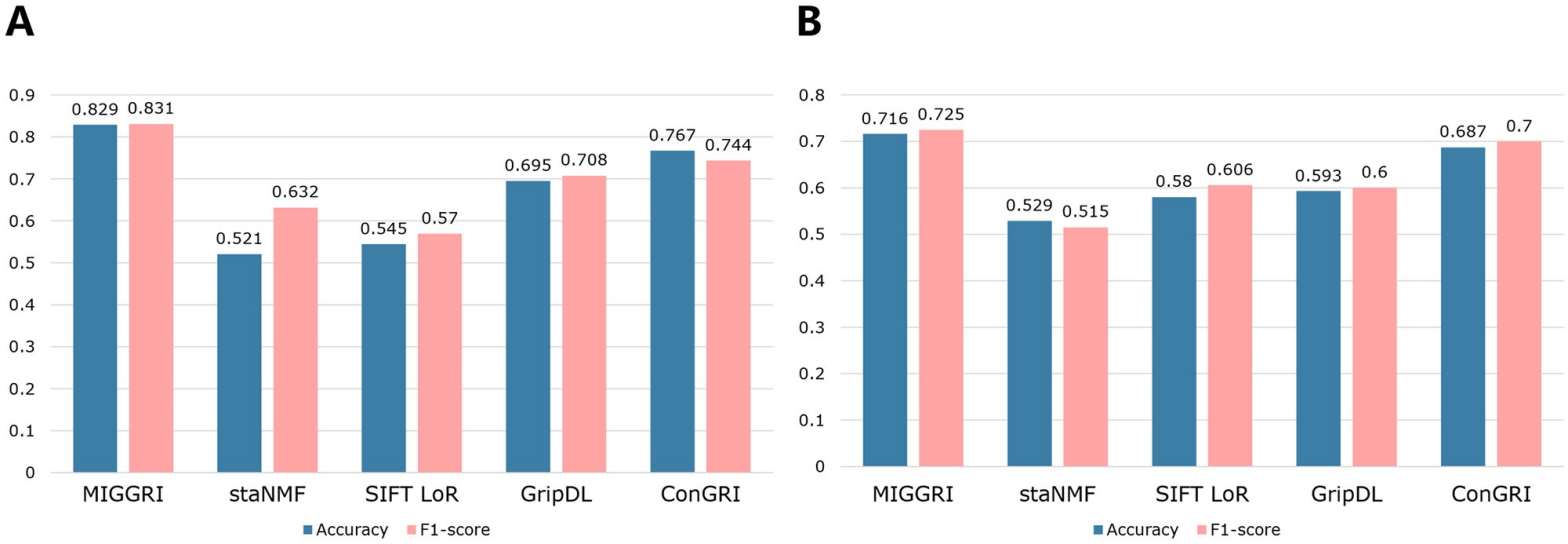
Performance comparison of GRN inference for *Drosophila* eye development (A) and mesorderm development (B).

The comparison results on the eye development dataset are shown in Fig 3A. As can be seen, supervised deep learning-based methods have great advantages over traditional algorithms, indicating that deep learning structures can exploit gene expression images and extract better features. Compared with the second-best model ConGRI, MIGGRI increases the accuracy by 6.2% and F_1_ by 8.7%. Among these models, the unsupervised method staNMF performs the worst because it identifies indirect gene-gene interaction based on similar components in the images rather than regulatory interaction. The traditional method SIFT_LoR also has very poor performance, indicating that the SIFT features are unable to reflect the expression patterns. ConGRI also adopts contrastive learning to extract image features while it uses fully connected MLP for classification. MIGGRI improves ConGRI with a multi-instance GNN leveraging the graph topological information, thus achieving the best prediction performance. The results suggest that the topological information improves the performance of inferring gene regulatory interaction.

The results from the experiment on the mesoderm development dataset are consistent with those of the eye development dataset, as shown in Fig 3B. Despite the mesoderm network being much smaller than the eye development network, MIGGRI still outperforms all other models, achieving an accuracy of 0.716 and an F_1_ score of 0.725. Notably, MIGGRI surpasses the second-best model, ConGRI, by 2.9% in prediction accuracy and 2.5% in F_1_ score. This highlights that the topology of a small graph can still enhance the node embeddings.

### Investigating the major components in MIGGRI

**Fig 4:**
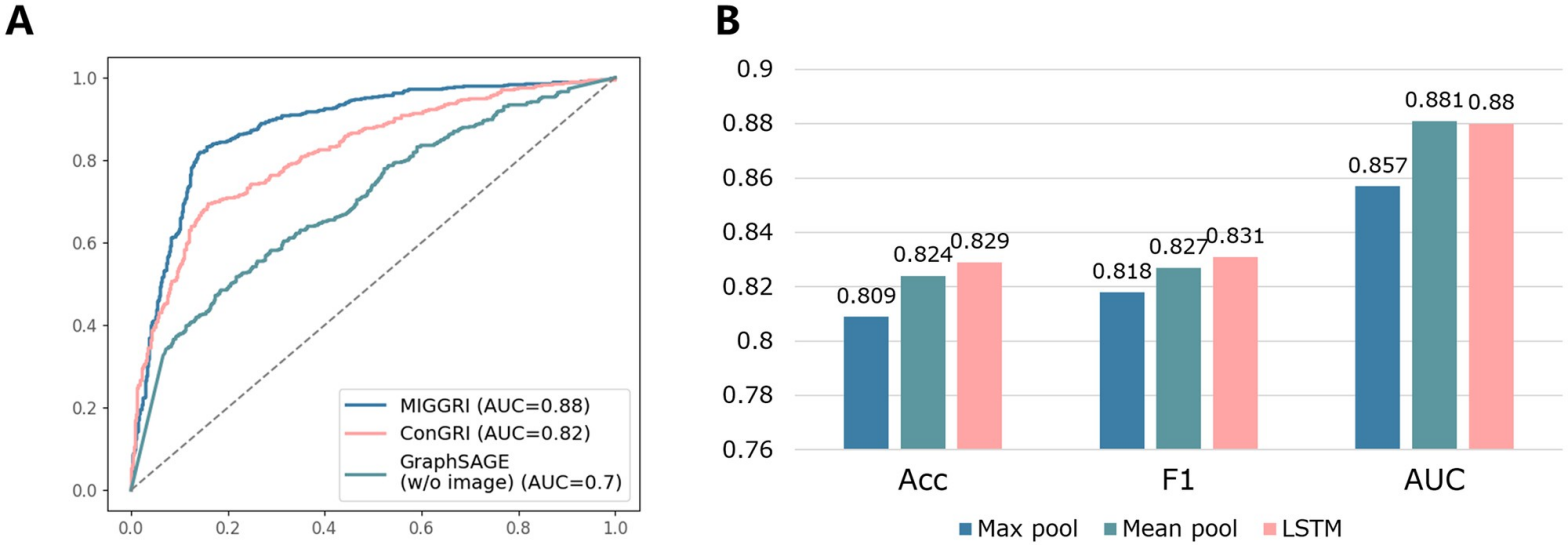
(A) ROC curves of MIGGRI, ConGRI and GraphSAGE (w/o image). (B) Comparison of three alternative aggregation modules in MIGGRI.

In addition to the comparison with the existing methods for inferring gene interactions, we also investigate the impact of the major components in MIGGRIby using the eye development dataset. To assess the importance of the topology of known GRN and the spatial image information, we compare the ROC Curves of MIGGRI, ConGRI, and GraphSAGE (w/o image). ConGRI also employs contrastive learning for image feature extraction. However, instead of using GNN, it concatenates the embeddings of two genes and feeds them into a multi-layer perceptron for prediction. GraphSAGE (w/o image) aims to provide baseline performance for assessing the contribution of the spatial expression data. Thus, this model has exactly the same graph structure and GNN model as those of MIGGRI while no spatial expression information is involved, i.e. all gene representations in the graph are randomly initialized in GraphSAGE (w/o image). As shown in Fig 4A, GraphSAGE (w/o image) benefits from the powerful learning ability of GNNs, which obtains an AUC (Area under the ROC Curve) of 0.70 by learning the network topological information. ConGRI adopts contrastive learning to extract image features and achieves an AUC of 0.82, outperforming the GraphSAGE (w/o image) by 0.12. Compared with the previous two models, MIGGRI reaches 0.88 on the AUC score, which has an obvious advantage over the other two methods. The results again show that topological information benefits the gene regulatory interaction inference, and suggest that both the proper representation of spatial expression data and the topological information are important for inferring gene regulatory interaction.

Then, we compare different aggregation modules. The aggregation module enables our method to handle the multi-instance input. As each gene is associated with a set of images and each image corresponds to an embedding vector, the aggregator aims to output a single vector for each gene, thus a simple pooling method can also be used. Here we compare max pooling, mean pooling, and the shuffled LSTM method (adopted by MIGGRI). The comparison results are shown in Fig 4B. The three aggregators all achieve over 0.8 accuracy. The mean pooling method performs better than max pooling, perhaps because the mean operator leads to more stable representations. In contrast to pooling-based methods, LSTM has learnable parameters and achieves the best overall performance.

### Predicting GRNs with missing data

**Fig 5:**
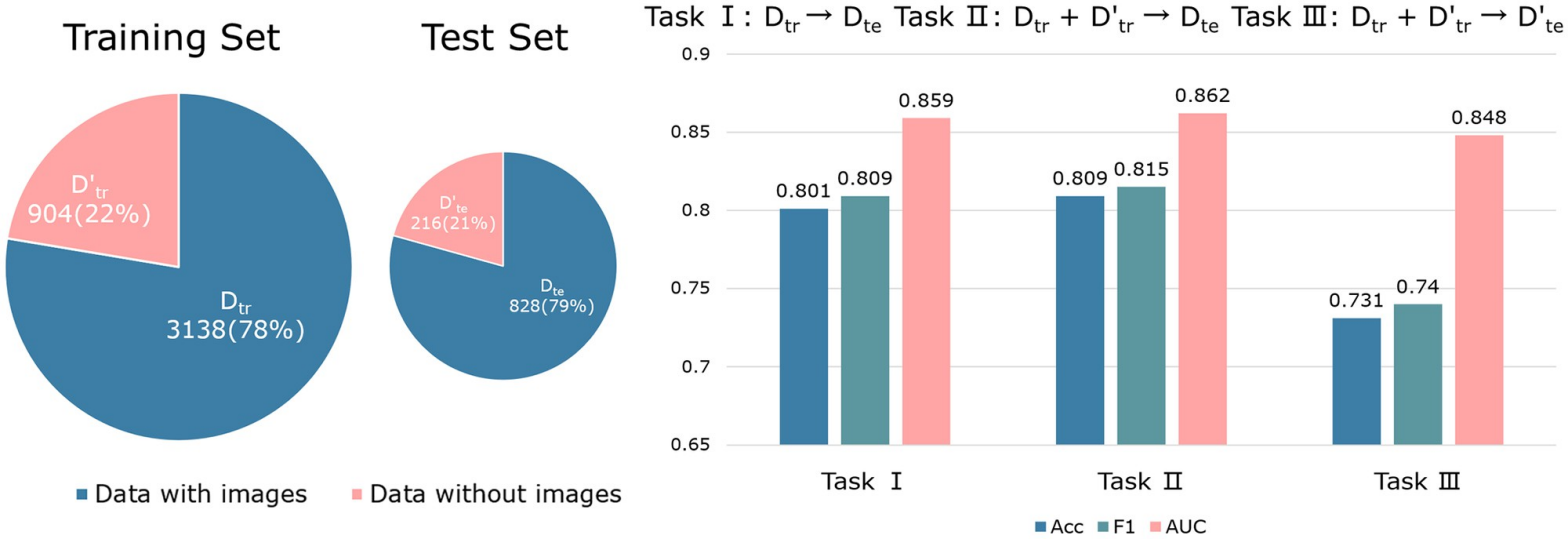
Results of GRN inference with missing data. The pie charts on the left show numbers of samples and percentages of the four datasets, where D_tr_ and D_te_ denote the training and test sets with image information, ${\text{D}}_{tr}^{\prime }$ and ${\text{D}}_{te}^{\prime }$ are the training and test sets without image information, and ‘→’ denotes fitting a mapping function from the training set (left) to the test set (right). Here the mapping function is learned by MIGGRI. The bar chart on the right shows the performance of three tasks.

As mentioned in the Introduction section, the missing data issue is very common in transcriptomics data. For instance, in our datasets, some genes may have expression images from only one or two orientations or even no image at all, while they are associated with regulatory interactions in the known GRN. Previous methods often discard genes with missing features, which limits their prediction capability. In contrast, GNN-based models can alleviate the reliance on input features by leveraging the relationships between genes captured by the graph structure. To evaluate the robustness of MIGGRI in scenarios with missing data, we conduct an experiment using a network with missing expression data. Specifically, we use 90% of the genes in the *Drosophila* eye development dataset, with their expression image features serving as input node features. For the remaining 10% of genes, we initialize their node features with random vectors following a normal distribution. We design three tasks for performance comparison. The task settings and their results are shown in Fig 5. Here we use mean pooling as the feature aggregator (the results of using other aggregators have the same trend and are given in S3 Table).

As can be seen, the accuracy of Task II is slightly higher than that of Task I. The experiments are repeated 20 times with random splits and we compute the statistical significance of the performance difference using pair-wise *t*-tests. We find that the accuracy difference is statistically significant at a 95% significance level, with a p-value of 0.036. This result suggests that adding genes without expression images can enhance the prediction accuracy for those genes with complete expression information. This finding demonstrates that link predictions in the graph can benefit a lot from the network topology, as the newly added genes introduce more links in the network and more data for training. The results of Task III demonstrate that MIGGRI can tune randomly initialized gene features into useful embeddings, achieving fairly good prediction accuracy (AUC 0.848 and F_1_ 0.740).

The capability of handling missing data is a great advantage of MIGGRI over previous GRN inference methods. Although the deep learning models, GripDL and ConGRI, achieve high accuracy on standard datasets, they are unable to handle the genes with no expression data, as they only use expression images as the input features. Therefore, MIGGRI has potentially much wider application in various GRN inference scenarios.

### Recovering the missing expression images

The experiment in the previous section shows how MIGGRI deals with the missing data issue, and we can see that the GNN-based model learns meaningful representation for the genes without spatial expression information. In this section, we further exploit the trained model to recover the missing expression images.

First, MIGGRI is trained on a network consisting of genes with complete information, then the nodes with randomized feature vectors are added to the network (the associated links of these nodes are also added). Second, we keep the model’s parameters unchanged and compute gradients of the loss function with respect to the feature vector of each newly added gene. Through the gradient descent, we optimize the representations of these randomly initialized gene nodes. In the third step, we use the inferred gene representations to recover spatial expression images.

**Fig 6:**
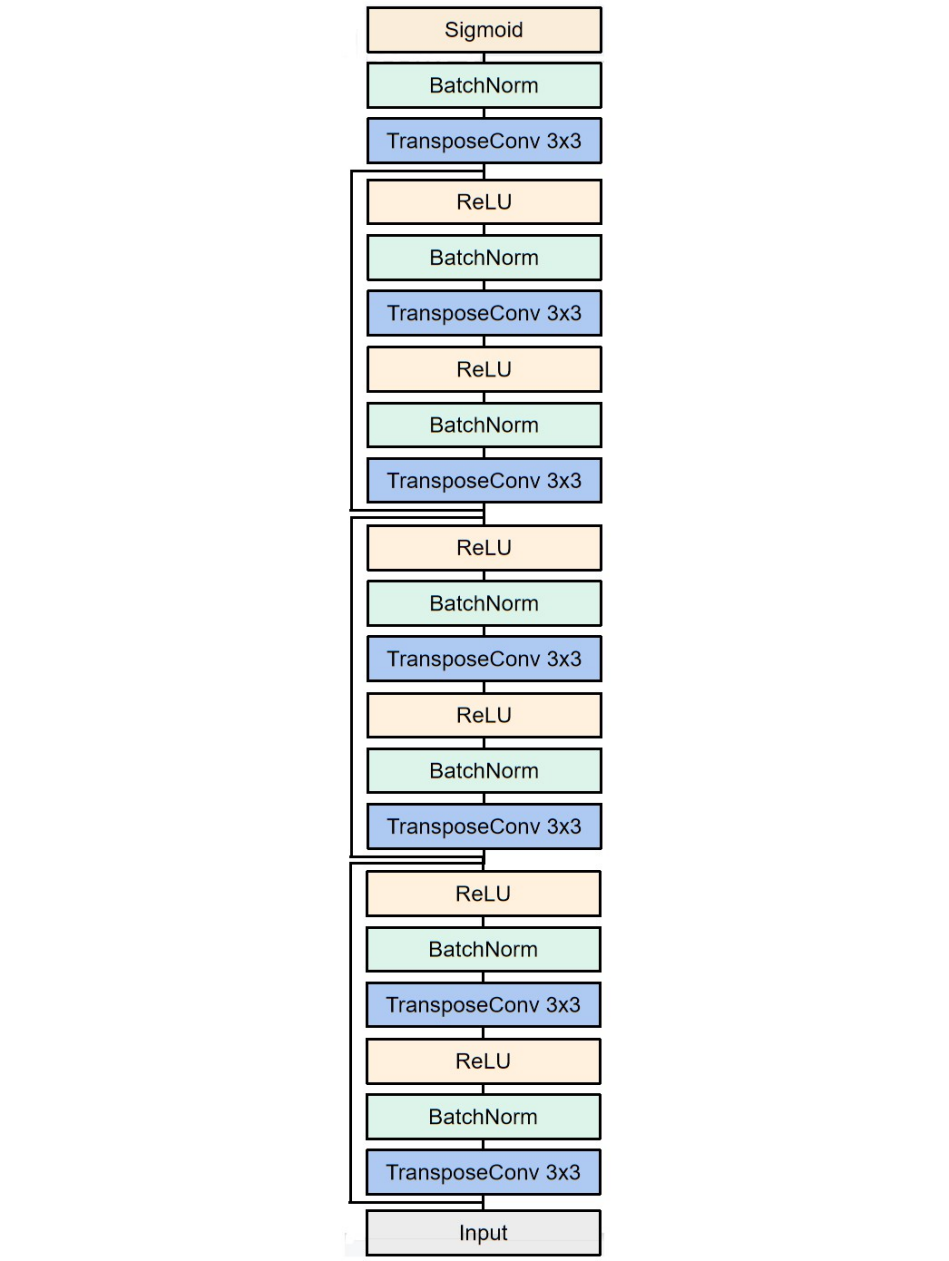
Architecture of the decoder.

To achieve this goal, we train a decoder, whose inputs and target outputs are the feature embeddings and expression images of the genes with complete information, respectively. The decoder’s structure is shown in Fig 6, which is a deconvolution network with residual connections. The reconstruction loss is the min-squared-error between the generated images and ground truth (GT) images. We use the Adam optimizer to train the decoder for 10 epochs with a batch size of 64. The learning rate is set to be 2e-4. In the experiment, 10% of the genes are left as the unseen genes, which are removed from the training graph and not used for training. After training, the model parameters are frozen and we add those unseen genes into the graph with randomly initialized features. Through gradient descent with respect to the feature vectors of the unseen nodes, we obtain updated representations for the unseen genes, which are fed into the trained decoder to yield the recovered images. As images from different orientations vary a lot, we train three decoders for the three orientations, respectively.

**Fig 7:**
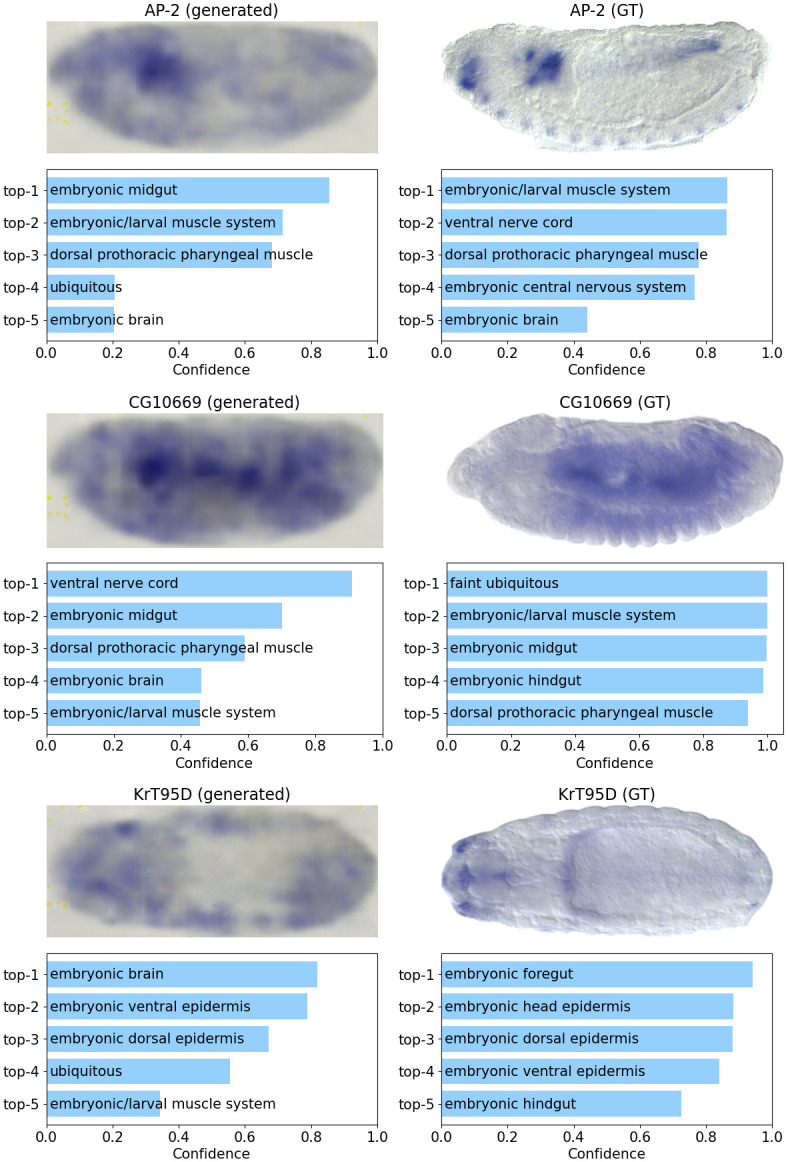
Top 5 predicted CV terms by Annofly for generated expression images (left) and ground truth (GT) images (right). The pixel intensity denotes expression level, and deep blue region means high expression region.

Fig 7 shows some examples of recovered images. Although the generated images have blurred texture, they retain some spatial distribution information of gene expression, especially the high-intensity regions. To evaluate the generation quality regarding the retained spatial distribution information in images, we use an annotation tool for *Drosophila* embryos, Annofly [36], that predicts anatomical and developmental terminology (In the BDGP database, each gene is annotated by some terms from a controlled vocabulary (CV), i.e. an ontology describing anatomical and developmental properties. The CV terms correspond to local regions in the images, such as ‘brain primordium’, ‘ventral nerve cord primordium’, and ‘head mesoderm PR’.) for spatial expression images. As can be seen from Fig 7, among the top 5 predictions, the predicted terms of generated images have a lot of overlap with those for the GT images. Take the gene *AP-2* as an example, there are 3 terms in common between the two top 5 lists, and the most confident term of GT image (‘embryonic/larval muscle system’) ranks the second for the generated image. *CG10669* and *KrT95D* have 3 and 2 common terms respectively between their generated and GT images. The overlap in the annotation terms suggests that the recovered images are semantically similar to the GT images. In other words, the generated features contain essential information that describes the functions and properties of genes.

### Predicting for unknown interactions

**Fig 8:**
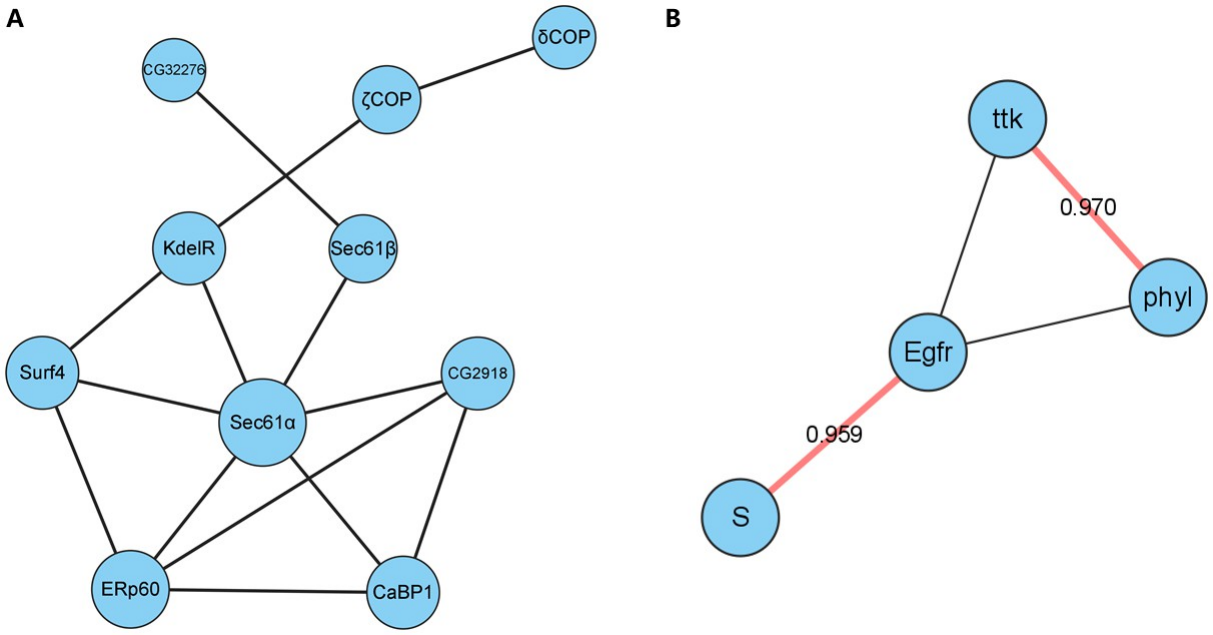
Two prominent sub-networks composed of links from the top 100 predicted interactions that are not in the known GRN. (A) A network participating in protein export and protein processing in the endoplasmic reticulum. (B) A sub-network of the EGFR pathway.

Given the gene representations yielded by the trained GNN, it is convenient to predict the existence of interaction between all gene pairs. Here we obtain the possibilities of interaction for all the unknown links in the graph of *Drosophila* eye development and analyze the top 100 ones by searching them in the FlyBase [37] and STRING [38] database to find supporting evidence of their regulatory relationship and the known pathways that they belong to. Especially, we find two prominent sub-networks. The first one is a network mainly composed of transmembrane proteins, as shown in Fig 8A. The Sec61 protein complex expressed by *Sec*61*α* and *Sec*61*β* genes in the network translocates secretory and membrane proteins into the lumen of the endoplasmic reticulum (ER), where they attain their correct three-dimensional structure. During this period, proteins undergo post-translational modification [39]. Both *CaBP1* and *ERp60* in the network are also located in ER and involved in the regulation of protein folding [40]. The widely conserved ER membrane protein complex (EMC) facilitates the biogenesis of a wide range of membrane proteins. For instance, ER membrane protein complex is required for the insertions of late-synthesized transmembrane helices of Rh1 in *Drosophila* photoreceptors [41]. Generally, the entire network is involved in protein export and protein processing in the endoplasmic reticulum. It constructs and influences Golgi-associated vesicle membranes and endoplasmic reticulum vesicular transporters, and is essential for *Drosophila* eye development [42, 43].

The second one belongs to the Epidermal Growth Factor Receptor (EGFR) signaling pathway [37], which mainly includes two regulatory interactions, i.e. *Egfr–S* and *ttk–phyl*, where *Egfr* and *ttk* are TFs. Both of the two links have predicted scores greater than 0.95. Fig 8B shows their interactions. The EGFR signaling pathway plays an important role in cell recruitment, ommatidium spacing, cell proliferation, and survival in *Drosophila* eye development. Both over-activation and under-activation of the EGFR signaling pathway can lead to ommatidial rotation defects [44, 45]. The EGFR signaling pathway is essential during eye development, and the expression of a dominant-negative EGFR completely prevents retinal formation [46]. Activation of the EGFR pathway recruits photoreceptor cells and cone cells in the *Drosophila* eye, and ED, a cell adhesion molecule, can inhibit EGFR signaling by regulating the activity of the *ttk* transcriptional repressor [47]. In addition, the degradation of the gene *ttk* through the gene *phyl* binds to the BTB domain of *ttk* during eye development [48, 49]. At the same time, the physical interaction between the gene *ttk* and the gene *phyl* has been verified in various experiments [49–51], and the gene *ttk* has an inhibitory gene regulation effect on the gene *phyl* [52].

In addition to the two networks discussed earlier, MIGGRI recognizes more interactions that form small functional networks (two examples are shown in S1 Fig). It is worth noting that, in addition to the high-confident interactions used for training from [22], MIGGRI predicts a number of interactions that are still under study. The study of [22] provides a larger number of medium-confident interactions than high-confident interactions, and we find that although some prediction results are not confirmed in the database and literature, they are present in the medium-confident interactions. Some of these predictions are listed in S6 Table. For instance, *Xbp*1 appears in two of the identified interactions, encoding a transcription factor that mediates the unfolded protein response. *Xbp*1 mutants fail to develop beyond the second instar larval stage, indicating a requirement to resolve inherent ER stress during normal development. *Su*(*H*) is predicted to interact with *Xbp*1. *Su*(*H*) is a key transcriptional regulator that has been shown to be tightly linked to processes such as eye development in *Drosophila*. Another gene predicted to interact with *Xbp*1 is *noc*, which has also been proven to be associated with *Drosophila* eye development [37]. Therefore, by cross-verifying the predicted potential interactions obtained by MIGGRI with the reported medium-confident ones, new discoveries of true interactions can be achieved.

What’s more, note that the negative pairs in our dataset are randomly sampled from the links that are not included in [22], while a few of them may be unrevealed interactions. We check the prediction results on the negative training data and find several links with high prediction scores. Then we search them in the STRING database [38] and find supporting evidence for some of them as listed in Table 1. For instance, *grh* and *vvl* participate in a regulatory network controlling epithelial maturation. Reduction of the POU domain TF Ventral veins lacking (Vvl) largely ameliorates the airway morphogenesis defects of *grh* mutants. Vvl and Grh proteins additionally interact with each other and regulate a set of common enhancers during epithelial morphogenesis [53]. In *Drosophila*,*ac* is a basic helix-loop-helix activator (bHLH A) gene which affects Su(H) and proneural bHLH A protein binding during Notch signaling [54]. Moreover, the *ac–sc* proneural genes are expressed in clusters of cells that prefigure the positions of each macrochaete, and *ara* is also involved in this process. The lack of *ara* will lead to the failure of expression [55].

**Table 1:** Gene regulatory interactions with high confidence on STRING.

Gene 1	Gene 2	Confidence score	Reference
*grh*	*vvl*	0.738	[53]
*ara*	*ac*	0.641	[55]
*Su*(*H*)	*ac*	0.628	[54]

**Fig 9:**
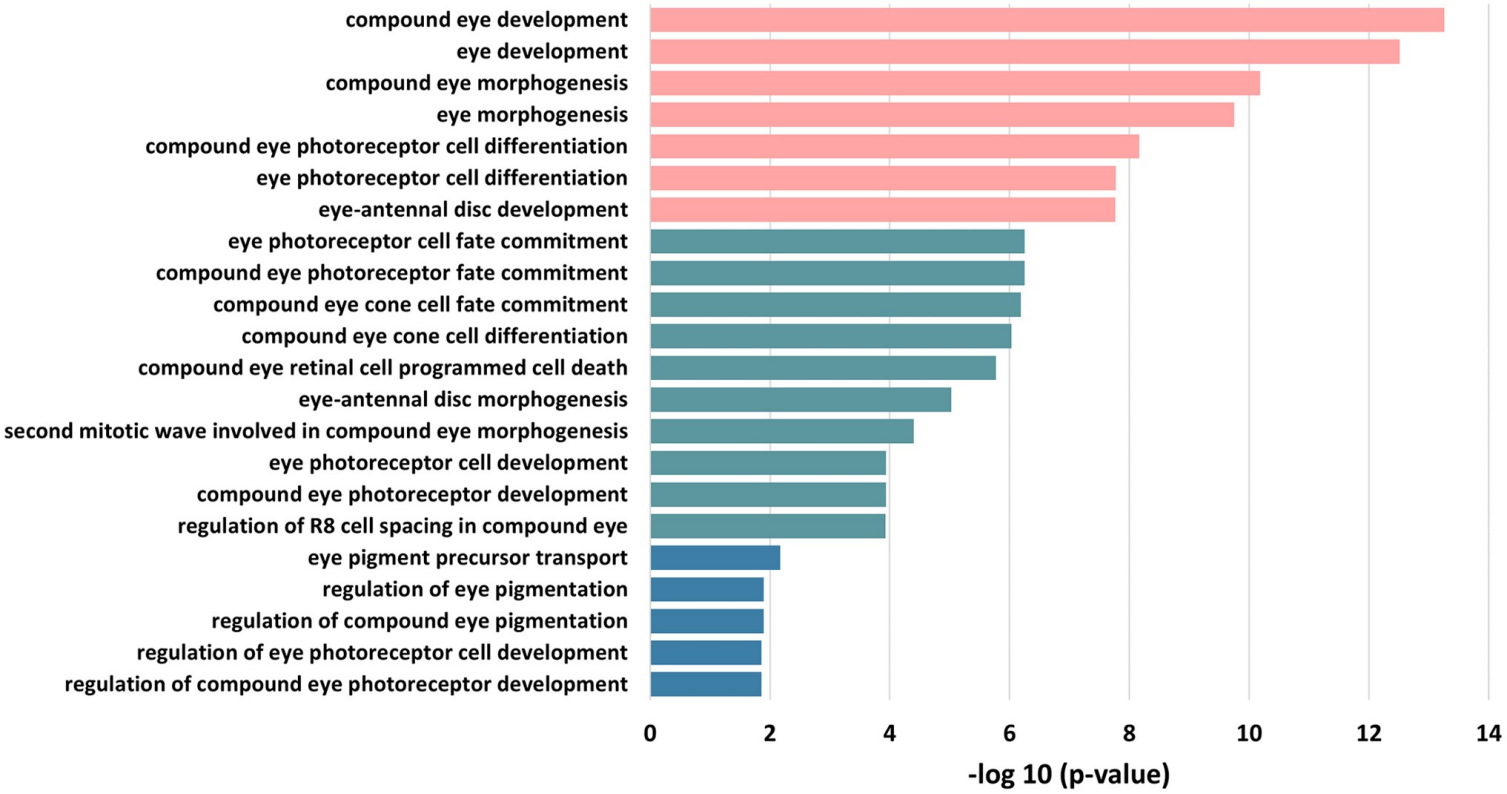
Enriched GO terms for the genes with links of predicted score over 0.95. The detailed information is provided in S5 Table.

In addition, we analyze the GO terms for all genes present in the predicted links with scores greater than 0.95, using BiNGO [56] in Cytoscape [57]. Fig 9 shows 19 biological process GO terms related to eye development, which are ranked by p-value (detailed values are showin in S5 Table). For example, the genes annotated by terms like ‘compound eye photoreceptor cell differentiation’ and ‘compound eye retinal cell programmed cell death’ involve in regulating the development of important components such as the retina and photoreceptor of eyes. Besides, there are many other terms not included in Fig 9, which denote basic cell activities, such as ‘regulation of transcription, DNA-dependent’. They are also essential for *Drosophila* eye development. Overall, a substantial proportion of the genes in the top predicted interactions are closely related to eye development.

### Interpreting the crucial subgraphs for prediction

The results of previous sections have shown that in MIGGRI, the judgment of the regulatory relationship between a pair of genes does not just depend on their own information but also on their neighborhood genes, which constitute a subgraph. In this section, we explore the biological significance of such subgraphs. Especially, we select the master regulator *grh*, a hub gene in the *Drosophila* eye development GRN with the largest degree, which can regulate a total of 197 target genes. The link *grh–dlp* is in the test set and successfully identified by MIGGRI. We apply the GNN-Explainer [58] (Details are described in Suppl. Materials) to find the links in the known graph that contribute to the prediction.

**Fig 10:**
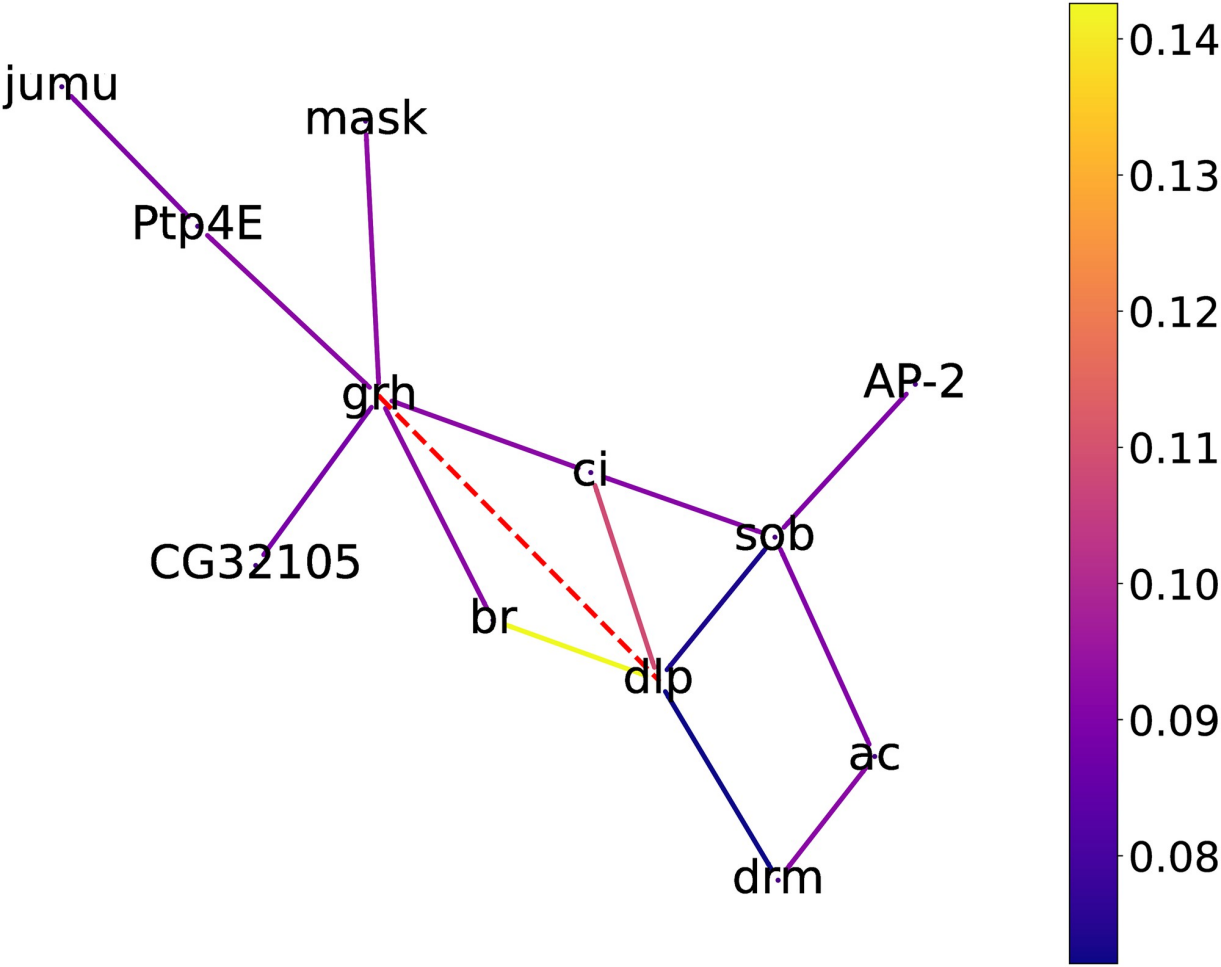
The heat map of a subgraph that determines the prediction for the interaction *grh–dlp* identified by GNN-Explainer. The red dashed line represents the interaction of interest, and the color of edges denotes their weights. For a clear visualization, we remove some edges with low weight.

Fig 10 shows the subgraph including the important links highlighted by GNN-Explainer for predicting *grh–dlp*, which includes 12 genes. By searching the FlyBase, we find that the genes *grh*, *dlp*, and *PtpE4* are all in the Fibroblast Growth Factor Receptor (FGFR) signaling pathway, which is an important pathway regulating cell morphogenesis involved in differentiation [59] and related to *Drosophila* eye development [60]. The importance level of *PtpE4–grh* ranks in the top 5.4% among the related interactions. We also search these three genes as a group in STRING and find that they function in cell morphogenesis involved in differentiation.

The consistency between the identified subgraph and known pathways suggests that MIGGRI can capture real regulation knowledge from GRN topology, which is a good interpretation for the learned model, i.e. the learned model could serve as a knowledge base. By applying an analysis tool, like GNN-Explainer, to a specific link in the network, a subgraph that determines the link can be recognized, which may facilitate the discovery of potential pathways.

## Discussion

We present a novel deep learning-based model, MIGGRI, for reconstructing gene regulatory networks (GRNs) in *Drosophila* eye development and mesoderm development. The proposed model has demonstrated superior performance compared to traditional methods and recent deep learning models, achieving substantial improvements in the reconstruction of GRNs.

The proposed model utilizes a contrastive learning scheme to generate high-quality image feature representations for genes. This feature extraction process is guided by known regulatory relationships and differentiates between interacting and non-interacting pairs. The quality of image embeddings relies on sufficient training at this stage, and it has a great impact on the final performance. Although contrastive learning mitigates the small data issue by generating numerous input image pairs for training, the model performance may be degraded for datasets with very few known interactions, such as the Mesoderm data. Nevertheless, when compared to methods that use single images as input, MIGGRI has a much higher capacity for handling small data.

As for the feature aggregation, the LSTM-based aggregator is adopted to generate a comprehensive representation for each gene from all image features that record their expressions. Since the LSTM is originally designed for sequence data, a random shuffling strategy is applied to ensure order invariance. We compare the randomly shuffled LSTM aggregator with two set-based aggregators, namely mean-pooling and max-pooling. The experimental results demonstrate that the randomly shuffled LSTM outperforms the other two aggregators. While we do not provide a further comparison with other aggregation methods in this paper, it is worth noting that it would be feasible to integrate attention-based or transformer-based aggregation methods into our proposed model. In our future work, we plan to explore additional aggregation methods to further improve the performance of our model.

The proposed MIGGRI framework exhibits outstanding performance on the *Drosophila* development datasets. However, the current study is limited by the availability of other large-scale gene regulatory networks and corresponding accessible spatial expression data. The BDGP database provides an abundance of spatial expression data for *Drosophila*, enabling comprehensive and large-scale study of GRNs. Additionally, *Drosophila* genes are extensively annotated as a model species, and the standardized expression images are relatively free of noise and artifacts. This facilitates more accurate identification of potential gene regulatory interactions and provides ideal conditions for assessing model performance and validating deep learning efficacy. Furthermore, using embryo images, the reconstructed images can be clearly observed, offering model interpretability that is not possible with complex mammalian tissues.

To assess the robustness of MIGGRI to noisy images, we conduct supplementary experiments by using raw images from BDGP for the reconstruction of eye development GRN. These raw images are much noisier than the standardized images, with multiple embryos or partial embryos appearing randomly in the image with varying angles (as illustrated in S2 Fig). We maintain the same dataset splits and other experimental settings as before and only change the input images to raw images from BDGP. The results show that all methods experience a decrease in performance. Specifically, MIGGRI achieves an accuracy of 75.3%, ConGRI 72.5%, and GripDL 67.6%. Despite the impact of noise on MIGGRI’s performance, it still outperforms ConGRI by 3.7% in accuracy, indicating that MIGGRI retains its advantages over other methods when handling noisy images.

Although the scope of this study is limited to *Drosophila* and ISH imaging data, the MIGGRI framework has the potential to be a versatile tool for gene regulatory network (GRN) inference in various organisms and expression data. The contrastive learning approach used in MIGGRI allows for the learning of image features related to gene regulatory patterns in an end-to-end manner, without incorporating specific features about the organism or image type. As more gene expression image databases become available, particularly with the emergence of spatial expression platforms such as Visium [61], CosMX [62], and MERSCOPE [63], MIGGRI can be applied to a wider range of spatial expression data and GRNs.

Compared to methods based on gene expression or TF-binding data, image-based GRN methods are still in their early stages, and their potential is yet to be fully explored. The following features have the potential to expand the range of application scenarios for MIGGRI.

- It has good prediction performance for genes without spatial expression data, and we can reversely generate their expression images from the trained model, which may provide clues on their expression distribution. Therefore, the existing knowledge on interacting gene pairs can be incorporated into the network and boost the performance of the whole network no matter whether their spatial expression data is given.
- It allows further mining on the subgraphs that may correspond to combinatorial regulation (as illustrated in Fig 10). Thus, the downstream analysis based on the model could provide new insight into the joint regulation of TFs and key network motifs.
- It can be easily applied to other GRNs and expression data obtained from a variety of high-throughput technologies, as the multi-instance GNN is a general framework whose node features can represent image embeddings, single-cell RNA-seq data, etc. Moreover, the model can also be adapted to node-level prediction tasks on GRNs, like identifying cancer driver genes, by replacing the dot product module with a classification module based on node embeddings.

## Conclusion

Recent advances in spatial transcriptomics have revolutionized gene regulation studies and enabled the extensive discovery of co-expression patterns distributed in tissues and organs. There is an urgent need of computational methods for GRN reconstruction based on spatial expression data alongside large-scale data generation. Although increasing efforts to use single-cell and spatial expression data have been made, most of the attempts deal with scalar- or vector-based gene expression levels instead of the whole picture of expression in space, due to the difficulty in integrating information from numerous high-dimensional microscopic images and regulatory networks into learning models.

This study attempts to provide an image-driven protocol for elucidating gene regulatory interactions and we present MIGGRI, a multi-instance GNN model for inferring GRNs from spatial gene expression images. In the experiments on large-scale benchmarks and an independent test set, MIGGRI achieves significant improvement compared to other reconstructing methods. With the increasing throughput and resolution of the imaging techniques of spatial transcriptomics, image-based approaches will be of paramount importance in elucidating gene expression patterns and regulatory events in various organs, tissues, as well as whole organisms. As more and more GRNs have been revealed, MIGGRI can help refine regulatory networks, impute missing links, and extend our understanding of complex gene regulation mechanisms.

## Supporting information

S1 Text Details about the link-prediction version of GNN-Explainer. (PDF)

S1 Table Data statistics of the two GRNs. (PDF)

S2 Table Numbers of image pairs used to train the siamese convolution network. (PDF)

S3 Table Prediction results of two aggregators on the datasets with missing data. (PDF)

S4 Table Comparison of different GNN backbones. (PDF)

S5 Table Enriched GO terms for the genes with links of predicted score over 0.95. (PDF)

S6 Table Interactions among the top 100 predicted results overlapped with the medium-confident interaction set reported in [22]. (PDF)

S1 Fig Two other prominent sub-networks. **A** The network participates in the modification of RNA of ribosomes and other organelles and can regulate regulatory proteins such as RNA helicases. If knocked out, some retinal constituent proteins will not be expressed [64]. Besides the development of *Drosophila*, the network is also involved in eye development in other organisms, such as retinal development and repair in rats and humans [65, 66]. **B** The network makes up and affects muscle proteins related to muscle development in the *Drosophila* eye.
(PDF)

S2 Fig Examples of raw expression images in BDGP. (PDF)
