# Supplementary material for: MIGGRI: A multi-instance graph neural network model for inferring gene regulatory networks for Drosophila from spatial expression images
Source: PLoS Comput Biol. 2023 Nov 8;19(11):e1011623. doi: 10.1371/journal.pcbi.1011623 (PMC10659162; doi:10.1371/journal.pcbi.1011623)
Supplement: S1 Text — (PDF) [file pcbi.1011623.s001.pdf]

S1 Text. Details about the link-prediction version of GNN-Explainer

GNN-Explainer [1] was originally designed for analyzing the node classification cases, though it can be transferred to link prediction cases. Here we implement the link prediction version of GNN-Explainer.

In our task, we focus on the contribution of interactions, so we learn an edge mask only. To explain an edge (u, v), the optimization objective is shown in Eq. (1),

$$\begin{aligned} \min_{\boldsymbol{M}} - \sum_{c=1}^{C} \mathbb{I}_{\{y=c\}} P\left(Y = y | \boldsymbol{G} = \boldsymbol{A} \cdot \sigma(\boldsymbol{M})\right) + \\ \beta_1 \cdot H(\sigma(\boldsymbol{M})) + \beta_2 \cdot \sum_{i,j}^{n} \sigma(m_{ij})^2. \end{aligned} \quad (1)$$

$H(\sigma(\boldsymbol{M}))$ is defined in Eq. (2),

$$\begin{aligned} H(\sigma(\boldsymbol{M})) = \sum_{i,j}^{n} \left[-\sigma(m_{ij}) \log \sigma(m_{ij}) - \right. \\ \left. (1 - \sigma(m_{ij}) \log\left(1 - \sigma(m_{ij})\right)\right], \end{aligned} \quad (2)$$

where $\boldsymbol{A}$ is the adjacent matrix of an n-node subgraph formed by nodes u, v, and their neighbors in k-hop range. $\boldsymbol{M}$ is an $n \times n$ matrix representing the learning mask ($[m_{ij}]_{n \times n}$), and σ is the sigmoid function. $\mathbb{I}_{\{condition\}}$ is the indicator function, which equals 1 when the condition is true. C is the number of class which equals 2 in binary classification task, and y is the label of the target edge. β_1 and β_2 are two regularization parameters. Slightly different from the objective function in [1], we change the L_1 regularization of $\sigma(\boldsymbol{M})$ to L_2 regularization, because we would like to see the contributions of more interactions, while L_1 regularization prefers to lead to a sparse result.

The mask is trained by 50 epochs with the Adam optimizer, and the learning rate is 0.1. β_1 is set to be 0.1 and β_2 is set to be 0.05. The importance scores are generated from $\sigma(\boldsymbol{M})$.

References

1. Ying Z, Bourgeois D, You J, Zitnik M, Leskovec J. GNNExplainer: Generating Explanations for Graph Neural Networks. In: Wallach HM, Larochelle H, Beygelzimer A, d'Alché-Buc F, Fox EB, Garnett R, editors. Advances in Neural Information Processing Systems 32: Annual Conference on Neural Information Processing Systems 2019, NeurIPS 2019, December 8-14, 2019, Vancouver, BC, Canada; 2019. p. 9240–9251.
