# Supplementary material for: MIGGRI: A multi-instance graph neural network model for inferring gene regulatory networks for Drosophila from spatial expression images
Source: PLoS Comput Biol. 2023 Nov 8;19(11):e1011623. doi: 10.1371/journal.pcbi.1011623 (PMC10659162; doi:10.1371/journal.pcbi.1011623)
Supplement: S1 Table — (PDF) [file pcbi.1011623.s002.pdf]

S1 Table. Data statistics of the two GRNs.

GRN		Eye	Mesoderm
TF gene #		95	1
Target gene #		1196	670
TF-target pairs	positive #	2650	335
	negative #	2649	335
	total #	5299	670
