# Supplementary material for: MIGGRI: A multi-instance graph neural network model for inferring gene regulatory networks for Drosophila from spatial expression images
Source: PLoS Comput Biol. 2023 Nov 8;19(11):e1011623. doi: 10.1371/journal.pcbi.1011623 (PMC10659162; doi:10.1371/journal.pcbi.1011623)
Supplement: S2 Table — (PDF) [file pcbi.1011623.s003.pdf]

S2 Table. Numbers of image pairs used to train the siamese convolution network

GRN	Image pairs	Ventral	Dorsal	Lateral	Total
	Positve #	5761	14698	24886	45345
Eye	Negative #	4957	14006	24392	43355
	Total #	10718	28704	49278	88700
	Positve #	519	1600	3932	6051
Mesoderm	Negative #	445	1286	3848	5579
	Total #	964	2886	7780	11630
