# Supplementary material for: MIGGRI: A multi-instance graph neural network model for inferring gene regulatory networks for Drosophila from spatial expression images
Source: PLoS Comput Biol. 2023 Nov 8;19(11):e1011623. doi: 10.1371/journal.pcbi.1011623 (PMC10659162; doi:10.1371/journal.pcbi.1011623)
Supplement: S3 Table — (PDF) [file pcbi.1011623.s004.pdf]

S3 Table. Prediction results of two aggregators on the datasets with missing data.

Aggregator	Training set	Test set	Acc	F_1	AUC
max	$\mathcal{D}_{tr}$	$\mathcal{D}_{te}$	0.780	0.788	0.847
	$\mathcal{D}_{tr} + \mathcal{D}'_{tr}$	$\mathcal{D}_{te} + \mathcal{D}'_{te}$	0.771	0.777	0.833
	$\mathcal{D}_{tr} + \mathcal{D}'_{tr}$	$\mathcal{D}_{te}$	0.792	0.794	0.853
	$\mathcal{D}_{tr} + \mathcal{D}'_{tr}$	$\mathcal{D}'_{te}$	0.692	0.717	0.768
LSTM	$\mathcal{D}_{tr}$	$\mathcal{D}_{te}$	0.804	0.811	0.882
	$\mathcal{D}_{tr} + \mathcal{D}'_{tr}$	$\mathcal{D}_{te} + \mathcal{D}'_{te}$	0.796	0.808	0.872
	$\mathcal{D}_{tr} + \mathcal{D}'_{tr}$	$\mathcal{D}_{te}$	0.808	0.817	0.886
	$\mathcal{D}_{tr} + \mathcal{D}'_{tr}$	$\mathcal{D}'_{te}$	0.751	0.768	0.822
