# Supplementary material for: MIGGRI: A multi-instance graph neural network model for inferring gene regulatory networks for Drosophila from spatial expression images
Source: PLoS Comput Biol. 2023 Nov 8;19(11):e1011623. doi: 10.1371/journal.pcbi.1011623 (PMC10659162; doi:10.1371/journal.pcbi.1011623)
Supplement: S4 Table — (PDF) [file pcbi.1011623.s005.pdf]

S4 Table. Comparison of different GNN backbones.

GNN model	Aggregator	Acc	F_1	AUC
GAT	Max pool	0.715	0.758	0.761
GCN	Max pool	0.722	0.758	0.756
GraphSAGE-mean	Max pool	0.809	0.818	0.857
GAT	Mean pool	0.726	0.758	0.788
GCN	Mean pool	0.744	0.774	0.804
GraphSAGE-mean	Mean pool	0.824	0.827	**0.881**
GAT	LSTM	0.751	0.769	0.830
GCN	LSTM	0.719	0.672	0.774
GraphSAGE-mean	LSTM	**0.829**	**0.831**	0.880
