# Supplementary material for: MIGGRI: A multi-instance graph neural network model for inferring gene regulatory networks for Drosophila from spatial expression images
Source: PLoS Comput Biol. 2023 Nov 8;19(11):e1011623. doi: 10.1371/journal.pcbi.1011623 (PMC10659162; doi:10.1371/journal.pcbi.1011623)
Supplement: S5 Table — (PDF) [file pcbi.1011623.s006.pdf]

S5 Table. Enriched GO terms for the genes with links of predicted score over 0.95.

GO description	Freq	Freq %	P-value
compound eye development	73	8.3	5.50E-14
eye development	75	8.6	3.09E-13
compound eye morphogenesis	58	6.6	6.53E-11
eye morphogenesis	60	6.8	1.78E-10
compound eye photoreceptor cell differentiation	32	3.7	6.81E-09
eye photoreceptor cell differentiation	32	3.7	1.69E-08
eye-antennal disc development	22	2.5	1.73E-08
compound eye photoreceptor fate commitment	18	2.1	5.58E-07
compound eye cone cell fate commitment	8	0.9	6.48E-07
compound eye cone cell differentiation	10	1.1	9.26E-07
compound eye retinal cell programmed cell death	7	0.8	1.68E-06
eye-antennal disc morphogenesis	14	1.6	9.47E-06
second mitotic wave involved in compound eye morphogenesis	6	0.7	3.97E-05
compound eye photoreceptor development	15	1.7	1.15E-04
regulation of R8 cell spacing in compound eye	5	0.6	1.18E-04
eye pigment precursor transport	3	0.3	6.77E-03
regulation of compound eye pigmentation	3	0.3	1.26E-02
regulation of compound eye photoreceptor development	4	0.5	1.40E-02

‘Freq’ and ‘Freq %’ denote the frequency and frequency percentage, respectively.
