# Supplementary material for: MIGGRI: A multi-instance graph neural network model for inferring gene regulatory networks for Drosophila from spatial expression images
Source: PLoS Comput Biol. 2023 Nov 8;19(11):e1011623. doi: 10.1371/journal.pcbi.1011623 (PMC10659162; doi:10.1371/journal.pcbi.1011623)
Supplement: S6 Table — (PDF) [file pcbi.1011623.s007.pdf]

S6 Table. Interactions among the top 100 predicted results overlapped with the medium-confident interaction set reported in [1].

Gene 1	Gene 2
$Hr46$	Lac
$Su(H)$	$Xbp1$
noc	$Xbp1$

References

1. Potier D, Davie K, Hulselmans G, Sanchez MN, Haagen L, Huynh-Thu VA, et al. Mapping Gene Regulatory Networks in Drosophila Eye Development by Large-Scale Transcriptome Perturbations and Motif Inference. Cell Reports. 2014;9:2290–2303.
