# Supplementary material for: MIGGRI: A multi-instance graph neural network model for inferring gene regulatory networks for Drosophila from spatial expression images
Source: PLoS Comput Biol. 2023 Nov 8;19(11):e1011623. doi: 10.1371/journal.pcbi.1011623 (PMC10659162; doi:10.1371/journal.pcbi.1011623)
Supplement: S1 Fig — A The network participates in the modification of RNA of ribosomes and other organelles and can regulate regulatory proteins such as RNA helicases. If knocked out, some retinal constituent proteins will not be expressed [64]. Besides the development of Drosophila, the network is also involved in eye development in other organisms, such as retinal development and repair in rats and humans [65, 66]. B The network makes up and affects muscle proteins related to muscle development in the Drosophila eye. (PDF) [file pcbi.1011623.s008.pdf]

S1 Fig. Two other prominent sub-networks. A The network participates in the modification of RNA of ribosomes and other organelles and can regulate regulatory proteins such as RNA helicases. If knocked out, some retinal constituent proteins will not be expressed [1]. Besides the development of *Drosophila*, the network is also involved in eye development in other organisms, such as retinal development and repair in rats and humans [2, 3]. **B** The network makes up and affects muscle proteins related to muscle development in the *Drosophila* eye.

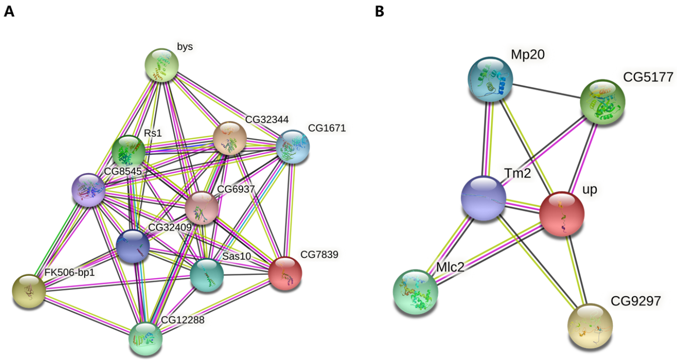

In addition, there are some interactions with prediction scores greater than 0.9 that also form functional networks. The network shown in S1A Fig is mainly involved in the modification of RNA of ribosomes and other organelles and can regulate regulatory proteins such as RNA helicases, which are related to the transcription and translation of nucleic acids. If knocked out, some retinal constituent proteins will not be expressed [1]. Besides the development of *Drosophila*, the network is also involved in eye development in other organisms, such as retinal development and repair in rats and humans [2, 3]. The network shown in S1B Fig can make up and affect muscle proteins related to muscle development in the *Drosophila* eye [4].

References

1. Lihong Z, A HK, Hwa KS, Apeksha T, S TR. Identification of genetic modifiers of TDP-43 neurotoxicity in Drosophila. PloS one. 2013;8.

2. Zeng H Yongand Qian, Campos MM, Li Y, Vijayasarathy C, Sieving PA. Rs1h$^{-/y}$ exon 3-del rat model of X-linked retinoschisis with early onset and rapid phenotype is rescued by RS1 supplementation. Gene Therapy. 2021;.

3. Xiao S, Sun W, Xiao X, Li S, Luo H, Jia X, et al. Clinical and genetic features of retinoschisis in 120 families with RS1 mutations. British Journal of Ophthalmology. 2021;.

4. Betz A, Lampen N, Martinek S, Young MW, Darnell JE. A Drosophila PIAS homologue negatively regulates stat92E. Proceedings of the National Academy of Sciences. 2001;98(17):9563–9568.
