# Supplementary figures and images for: MIGGRI: A multi-instance graph neural network model for inferring gene regulatory networks for Drosophila from spatial expression images
Source: PLoS Comput Biol. 2023 Nov 8;19(11):e1011623. doi: 10.1371/journal.pcbi.1011623 (PMC10659162; doi:10.1371/journal.pcbi.1011623)

**S2 Fig.** Examples of raw expression images in BDGP.

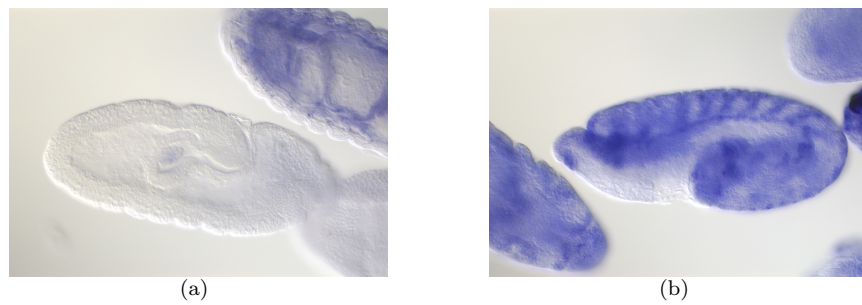

Supplement: S2 Fig — (PDF) [file pcbi.1011623.s009.pdf]
